# Lyophilization of Adeno-Associated Virus Serotypes for Storage and Global Distribution

**DOI:** 10.3390/biomedicines14010025

**Published:** 2025-12-22

**Authors:** Erin B. McGlinch, Haley E. Mudrick, Christopher H. Evans, Michael A. Barry

**Affiliations:** 1 Virology and Gene Therapy Graduate Program, Rochester, MN 55905, USA; mcglinch.erin@mayo.edu; 2 Molecular Pharmacology and Experimental Therapeutics Graduate Program, Rochester, MN 55905, USA; hmudrick11@gmail.com; 3 Departments of Physical Medicine & Rehabilitation, Orthopedic Surgery and Molecular Medicine, Mayo Clinic, 200 First Street SW, Rochester, MN 55905, USA; evans.christopher@mayo.edu; 4 Department of Internal Medicine, Division of Public Health, Infectious Diseases, and Occupational Medicine, Department of Immunology, Department of Molecular Medicine, Mayo Clinic, Rochester, MN 55905, USA

**Keywords:** gene therapy, adeno-associated viral vectors, storage, lyophilization, distribution

## Abstract

**Background**: Adeno-associated viruses (AAVs) are widely used vectors for in vivo gene therapy, but their standard storage at −80 °C limits deployment in regions lacking ultracold infrastructure. Strategies enabling stable AAV storage at higher temperatures are needed to support global distribution. **Methods**: Nine AAV serotypes were lyophilized in simple sucrose-based buffers. Post-lyophilization vector integrity was assessed by measuring in vitro transduction efficiency using a luciferase reporter in cell-based assays. Stability of selected serotypes (AAV2.5 and AAV6) was further evaluated over 8 weeks under varying storage temperatures. **Results**: Lyophilization in sucrose preserved transduction activity for all tested serotypes (AAV1, AAV2, AAV2.5, AAV3, AAV4, AAV5, AAV6, AAV8, AAV9, and AAVrh10). Notably, AAV2, AAV2.5, and AAV6 exhibited 3- to 6-fold increases in transduction, an effect attributable to the sucrose excipient rather than the lyophilization process itself. Long-term stability of lyophilized vectors varied by serotype, temperature, and vial-seal integrity. AAV6 retained full activity for at least 8 weeks when stored at 4 °C or −20 °C. **Conclusions**: AAV vectors can be effectively lyophilized in simple sucrose solutions, enabling storage at standard −20 °C freezer temperatures while maintaining functional activity. Optimization of lyophilization buffers and excipients may further extend AAV stability at higher temperatures, improving feasibility for global gene therapy deployment.

## 1. Introduction

**Figure 1:**
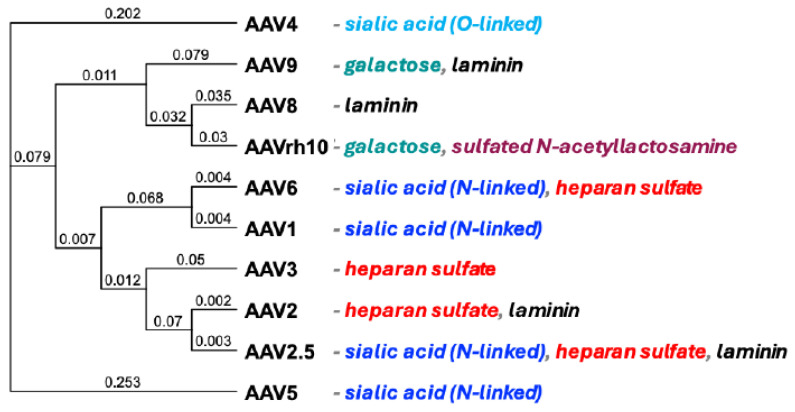
Phylogenetic tree of AAVs. AAV serotypes and the primary known cellular receptors utilized beyond the AAVR found in the literature [8,12,28,29,30,31]. Distance is uncorrected (“p”) with gaps distributed proportionally.

Adeno-associated viruses (AAVs) are promising vectors for gene therapy [1,2,3]. They are non-enveloped viruses from the *Parvoviridae* family with a single-stranded DNA genome [4,5]. These viruses are classified into different serotypes (e.g., AAV1, AAV2, and AAV5) and variants, each with unique capsid structures influencing receptor binding and cellular entry [6,7,8,9,10,11]. Each serotype mediates initial attachment and internalization by targeting different single or multiple receptors on different cell types [5,12] (Figure 1). These differences in receptor utilization lead to a variety of vector tropisms for different gene therapy applications.

While AAV vectors hold great promise to deliver gene therapy to the masses, they are complex bio-nanoparticles and are less stable than small-molecule drugs [13,14], despite being made up of only protein and DNA [4]. DNA and protein both may decrease stability at room or body temperature [13,15]. Storing AAV at higher temperatures is possible, but more vector activity is lost with increasing temperature.

The current preferred storage condition for AAV vectors is at −80 °C. This requirement to store AAVs at ultralow −80 °C temperatures can be challenging, even for clinics in the United States and other developed countries, even if they have good cold chains, since most clinical pharmacies do not have ultralow −80 °C freezers. This cold storage requirement makes it almost impossible to consider global gene therapy, since less-developed sites might not have 4 °C refrigeration, let alone −80 °C freezers. However, cold-chain limitations are only one of several barriers to implementing gene therapies in settings with fewer resources, alongside challenges related to cost, diagnostic infrastructure, and access to necessary supportive care.

For these reasons, there is great interest in improving the stability of AAV drugs [16,17,18]. Lyophilization, or freeze-drying, is one approach that might help achieve this goal [16,19,20,21]. Lyophilization involves freezing a drug in solution and then sublimating the water ice out of the frozen state under reduced pressure, converting it into a dry, solid state [19,22]. This technique prevents the formation of large ice crystals that can damage DNA, protein, and viral capsids [10,23]. Cryoprotectants and stabilizers can be added to aqueous buffers to enhance stability and protect against reductions in pH during lyophilization [19]. This can help ensure that vectors retain their infectivity and transduction efficiency after reconstitution [24,25].

To date, lyophilization has been tested for a subset of AAV serotypes, including AAV2 [19,26], AAV5 [27], AAV8 [15,22,24], and AAV9 [21]. This prior art demonstrates that lyophilization is possible for different AAVs. However, most studies examine serotypes in isolation, so it is unclear to what degree different serotypes with different biologies behave during lyophilization. Furthermore, it is unclear to what degree the methods for each AAV serotype must be fine-tuned, since each study applied somewhat different methods and formulations. Given these uncertainties, this study compared the durability of different popular AAV serotypes using a unified method of lyophilization using a simple lyophilization buffer that could be used easily across the globe.

## 2. Materials and Methods

### 2.1. Generation of Phylogenetic Tree

AAV serotypes were compared using MacVector 18.8.1 using ClustalW alignment (MacVector, Inc, PO Box 1147, Apex, NC, USA) using Neighbor Joining, Best Tree, and tie breaking = Systematic. Distance is uncorrected (“p”).

### 2.2. AAV Production and Titration

AAV vectors were produced using methods adapted from a previous paper [32]. The 293T cells were cultured in Dulbecco’s minimal essential medium (DMEM) with 10% fetal bovine serum (FBS) and 1% penicillin/streptomycin (P/S). Ten 150 mm dishes were triple-transfected with adenovirus pHelper plasmid, each AAV pRep2/CapX plasmid, and pAAV-GFP-Luc plasmid using polyethyleneimine (PEI). A total of 75 µg of pHelper and pRepCap were mixed with 150 µg of pAAV-GFP-Luc for a ratio of 1:1:2 and 2 mL of 1 mg/mL PEI (CAS No. 9002-98-6; Thermo Scientific Chemicals, Waltham, MA, USA) and were incubated at room temperature for 15 min. The transfection cocktail was then added dropwise to plates. Cell culture media was changed 24 h after transfection. The cells were harvested at 72 h after transfection by scraping and concentrated down to 17.5 mL by centrifugation at 250× *g* for 15 min at 4 °C. The cell pellets and 17.5 mL of remaining media were freeze–thawed 3 times in ethanol and dry ice. Virus was purified by a discontinuous iodixanol gradient marked with phenol red by centrifugation at 62,500 rpm for 2 h at 15 °C with a Beckman Type 70 Ti Fixed-Angle Titanium Rotor (Beckman Coulter, Brea, CA, USA). The 40% iodixanol band was collected by a 21-gauge needle and mixed with 5 mL phosphate-buffered saline (PBS). AAV particles were concentrated by centrifugation at 3700× *g* for 20 min at 15 °C in Amicon^®^ Ultra-15 Centrifugal Filter Units (Millipore Sigma, Burlington, MA, USA).

AAV vector genomes (vg) were quantified by qPCR using SYBR Green and the protocol developed by AddGene [33]. Probes targeting the AAV2 ITR were utilized (forward: 5′-GGAACCCCTAGTGATGGAGTT, reverse: 5′-CGGCCTCAGTGAGCGA). A standard curve was calculated on the same plate using a pTRS-Cre plasmid. The protocol was set as follows: 98 °C 3 min/98 °C 15 s/58 °C 30 s/read plate/repeat 39x from step 3/melt curve.

### 2.3. Lyophilization

**Figure 2:**
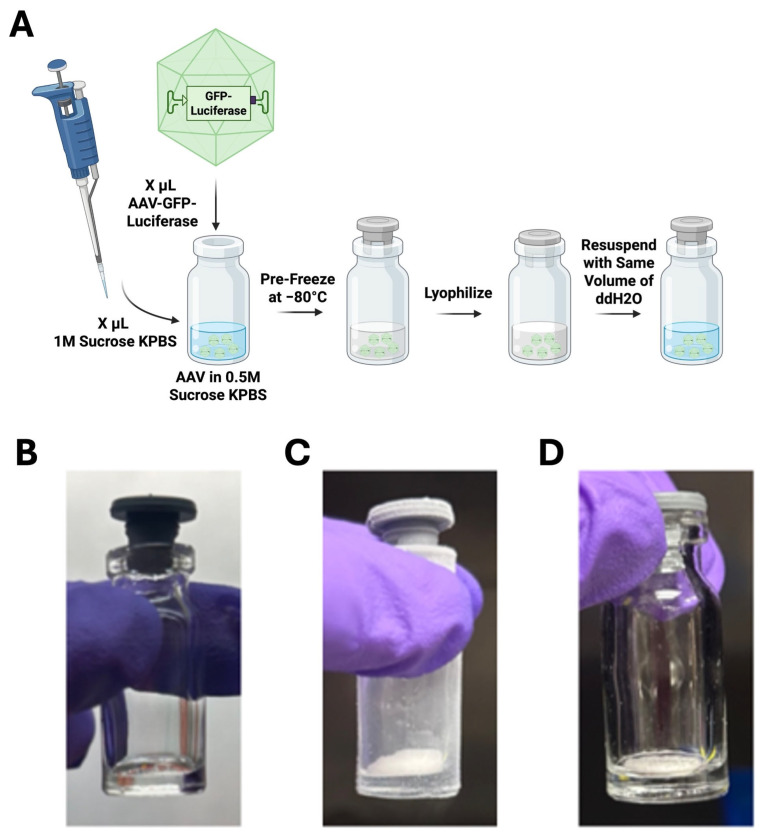
Schematic of lyophilization workflow. (**A**) Schematic of in vitro lyophilization experiments throughout the paper. For each experiment, the volume of virus needed to infect one well of a 96-well plate (seeded to a density of 4e4 cells/well) at a multiplicity of infection (MOI) of 1e4 vg/cell was calculated. This volume of virus (X uL) was added to a lyophilizer vial with an equivalent volume of 1M Sucrose KPBS buffer (or other buffer, as specified in experimental details) to make a final sucrose concentration of 0.5 M (or other, as specified). The sample was frozen at −80 °C for 15 min prior to being put in the lyophilizer, then it was lyophilized inside the glass vial. Before infecting a well, the sample was reconstituted in ddH_2_O to the original (pre-lyophilization) sample volume (in the example, 2X uL), and the reconstituted virus was added to the well. Virus controls consisted of non-lyophilized virus and 1 M Sucrose KPBS buffer in the same volumes as for the lyophilized samples (in the example, X mL each of virus and buffer), producing a 0.5 M sucrose concentration. This non-lyophilized 1:1 mixture of virus:buffer (in the example, 2X uL) was used to directly infect the well. Images of 0.5 M sucrose KPBS AAV2.5GL samples are shown (**B**) pipetted into the vial in liquid form, (**C**) frozen at −80 °C, and (**D**) lyophilized, prior to reconstitution in ddH_2_O (created in BioRender. Mcglinch, E. (2025) https://BioRender.com/879kdln).

Samples were lyophilized in the FreeZone^®^ TriadTM Freeze Dry System, Model 74000 Series (Labconco, Kansas City, MO, USA). Samples were lyophilized via a step-up protocol described in Table 1 [19], all at 30 mTorr. Samples were mixed at a 1:1 ratio of virus to lyophilization buffer (1 M sucrose, potassium phosphate-buffered saline (KPBS)) for an ending buffer of 0.5 M sucrose in 10 mM KPBS and pipetted into lyophilizer vials [Labconco 2 mL Glass Serum Bottle] as droplets. Vial caps were placed on top of the vials to allow air and temperature flow into the vial, but also enable automated capping of the contents under vacuum at the end of the process (Figure 2). AAVs were lyophilized at 30 mTorr, and the vials were capped under vacuum to avoid moisture contamination of the product.

The long-term stability experiment added Poloxamer 188 at 0.01% wv to the buffer for all conditions and controls as a surfactant to prevent adhesion to the glass. Vial caps were perched on top of vials to allow airflow. Lyophilizer vials were capped inside the lyophilizer under 30 mTorr vacuum to avoid moisture contamination.

### 2.4. Luciferase Assay

In vitro transduction was assessed by luciferase assay according to the provided protocol with the Bright-Glo^TM^ Luciferase Assay System (Promega, Madison, WI, USA). For the 96-well assays, 100 ul of the BrightGlo Reagent was added directly to each well, the plate was incubated for 3 min at room temperature, and then luminescence was read using a luminometer. Unless indicated otherwise, 293T cells were utilized.

### 2.5. Animals

Male adult FVB mice (Charles River Laboratories) were housed in the Mayo Clinic Animal Facility, and all animal handling and experiments were performed according to the provisions of the Animal Welfare Act, PHS Animal Welfare Police, the principles of the NIH Guide for the Care and Use of Laboratory Animals, and the policies and procedures of the Mayo Clinic. This study was conducted in the Mayo Clinic’s AAALAC (Association for the Assessment and Accreditation of Laboratory Animal Care)-accredited facilities and was approved by the Institutional Animal Care and Use Committee (IACUC). A total of 5 animals were randomly assigned to each group and were given one treatment per quadricep intramuscularly.

### 2.6. IVIS Imaging

Mice were anesthetized with isoflurane prior to receiving an intraperitoneal injection of 1.5 mL D-luciferin [20 mg/mL; Molecular Imaging Products, Bend, OR, USA]. Mice remained anesthetized for 10 min after intraperitoneal injection to allow full circulation of D-luciferin. Mice were then imaged in the IVIS^®^ Lumina X5 Imaging System (PerkinElmer, Waltham, MA, USA) under continued isoflurane anesthesia.

### 2.7. Statistical Analysis

All statistical analyses were performed using Prism 10 Graphical software.

## 3. Results

### 3.1. A Diverse Array of AAV Serotypes Survive Lyophilization

A panel of various AAV serotypes that target different receptors was selected for testing (Figure 1). The AAV serotypes tested were 1, 2, 2.5, 3, 4, 5, 6, 8, 9, and rh10. Each serotype was used to package single-stranded AAV vectors expressing the green fluorescent protein-luciferase (GFP-Luc) fusion protein, and each was stored at −80 °C in standard phosphate-buffered saline (PBS) buffer.

**Figure 3:**
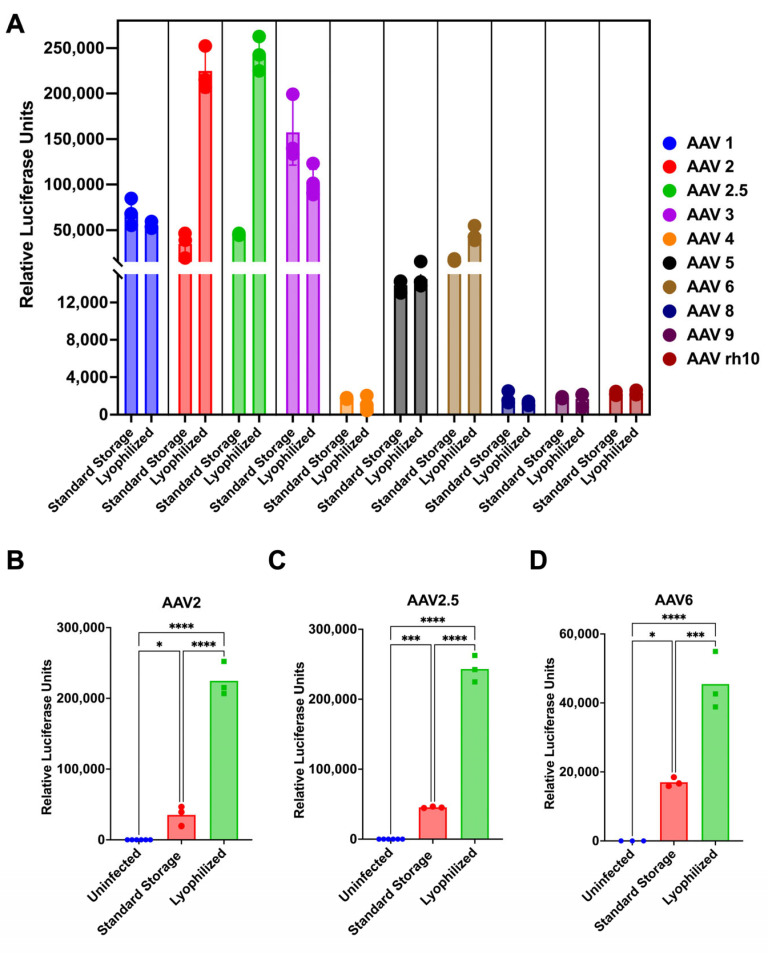
AAV serotypes and their transduction efficiency after lyophilization. AAV serotypes 1, 2, 2.5, 3, 4, 5, 6, 8, 9, and rh10, all expressing GFP-Luciferase transgene, were combined in a 1:1 mixture of 1 M sucrose KPBS buffer to virus and lyophilized. Lyophilized virus, compared with non-lyophilized virus freshly thawed from the −80 C freezer (standard storage), was used to infect 293T cells in triplicate at a multiplicity of infection (MOI) of 10,000 vg per cell, and (**A**) luciferase expression was assessed at 72 h post-infection via luciferase assay for all serotypes. Data is shown separately for the AAVs with significant transduction activity increase: (**B**) AAV2, (**C**) AAV2.5, and (**D**) AAV6. Error bars represent standard deviations (**** = *p* < 0.0001, *** = *p* < 0.001, and * = *p* < 0.05 by one-way ANOVA).

Following lyophilization, aliquots of the viruses were immediately reconstituted with water and were used to infect 293T cells in triplicate in 96-well plates at a multiplicity of infection (MOI) of 10^4^ viral genomes (vg) per cell. Under these conditions, none of the nine AAV serotypes lost any significant transduction efficiency after being lyophilized (Figure 3A). AAV serotypes 1, 5, 8, 9, and rh10 all exhibited no change in transduction efficiency with lyophilization. AAV3 and 4 experienced moderate, but statistically insignificant, decreases in transduction efficiency after lyophilization. Due to all AAV serotypes being produced in different batches in an academic research setting, no comparisons were made between transduction efficiencies between serotypes, as production can be highly sensitive to variations in upstream parameters in production, leading to potential inconsistencies in infectivity and functional performance [34].

### 3.2. The Transduction Activity of a Subset of AAV Serotypes Increases After Lyophilization

Unlike the other serotypes, AAV2, AAV2.5, and AAV6 all had significant increases in luciferase expression, indicating greater transduction efficiency after lyophilization (Figure 3B–D). AAV2 increased 6-fold, AAV2.5 increased 5-fold, and AAV6 increased 3-fold after lyophilization. To confirm this was not an occurrence only seen in 293T cells, it was confirmed in Chinese hamster ovary (CHO) cells (Figure S1).

### 3.3. Increased Transduction Activity After Lyophilization Is Mediated by Sucrose Excipient

**Figure 4:**
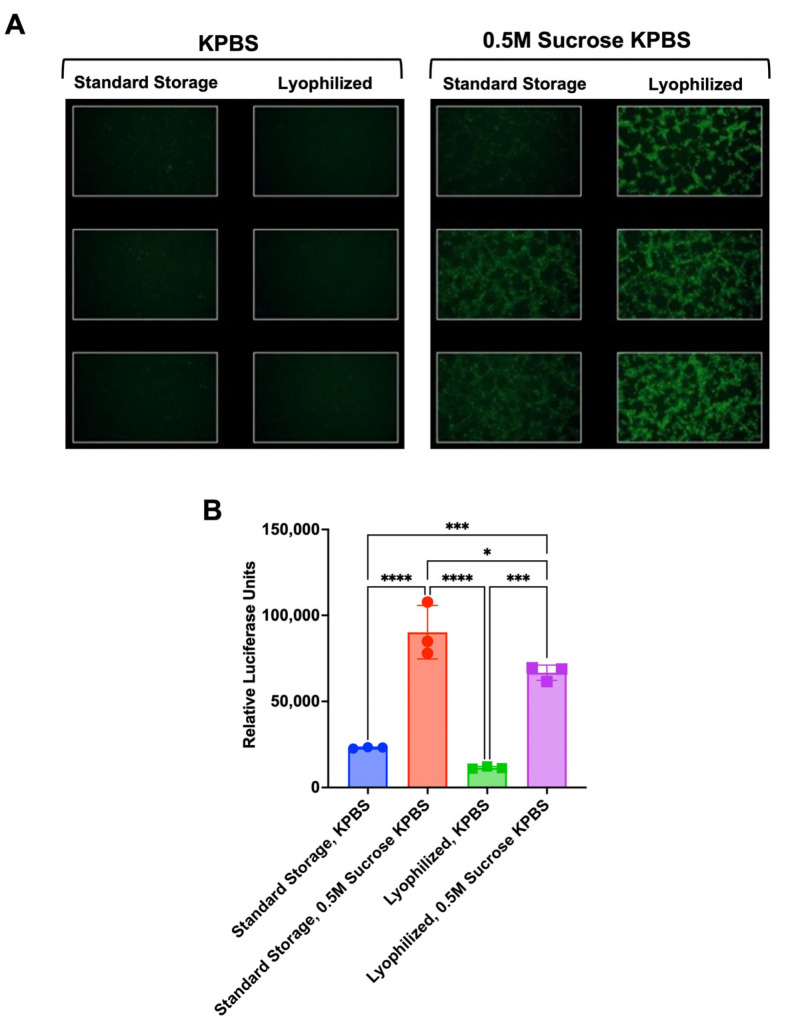
AAV2.5 transduction activity benefits from a buffer of 0.5 M sucrose. 293Ts were infected in triplicate with 10,000 vg per cell of AAV2.5-GFP-luciferase taken from standard storage (−80 °C) or lyophilized in KPBS buffer or 0.5 M sucrose KPBS. (**A**) GFP signal was assessed at 24 h post-infection via light microscope. Images obtained using 10× objective. (**B**) Luciferase signal was assessed via luciferase assay at 24 h post-infection. Error bars represent standard deviations (**** = *p* < 0.0001, *** = *p* < 0.001, and * = *p* < 0.05 by one-way ANOVA).

To elucidate whether the increase in transduction efficiency observed was a result of the lyophilization process or the lyophilization buffer, AAV2.5 frozen in PBS or AAV2.5 lyophilized in 0.5 M sucrose in 10 mM potassium phosphate (KPBS)-buffered saline were each thawed, reconstituted, and then mixed 1:1 with KPBS or KPBS with 1 M sucrose (for an overall 0.5 M sucrose concentration) and used to infect 293T cells (Figure 4). After 24 h, GFP and luciferase signals were measured by fluorescence microscopy and by luciferase assay. Under these conditions, 293T cells transduced with frozen or lyophilized AAV2.5 without added sucrose had weaker GFP activity when compared to cells that were supplemented with sucrose (Figure 4A). When luciferase activity was quantified, sucrose added to the cells increased transduction by virus taken directly from standard storage at −80 °C; transduction efficiency was further increased when using virus that had been lyophilized in the presence of sucrose (Figure 4B).

### 3.4. Sucrose Increases AAV Transduction After Viral Entry

We hypothesized that sucrose might be affecting the structure of certain AAV serotypes or their interactions with cellular receptors. If so, we would expect to see the sucrose effect occur in the first 30 min of an infection. An alternate hypothesis was that sucrose might be affecting cellular trafficking or metabolism after entry. If so, one might predict its effects would occur at later time points.

**Figure 5:**
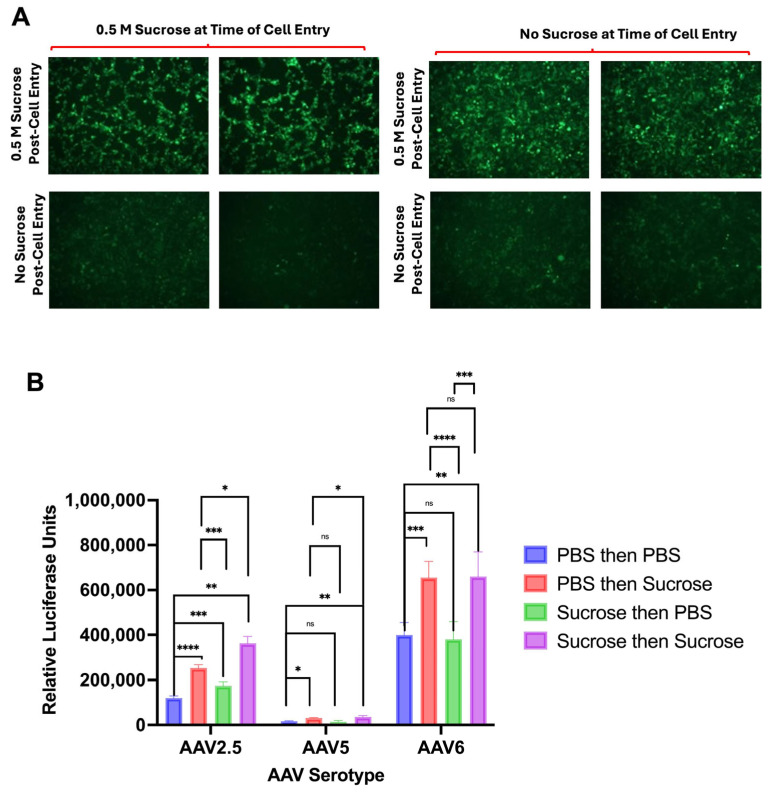
Sucrose affects AAV2.5 activity primarily after cellular entry. AAV2.5-GFP-Luciferase in either 0.5 M sucrose KPBS or KPBS alone was added to infect 293T cells in triplicate at a multiplicity of infection (MOI) of 10,000 vg per cell. After a 30 min 37 C incubation, the media and virus were removed from cells and replaced with either DMEM + sucrose (100 ul of 0.5 M KPBS + sucrose) or DMEM + KPBS (100 ul KPBS). Cells were then imaged at 48 h post-infection (images obtained using 10X objective) (**A**), and a BrightGlo luciferase assay was run at 48 h post-infection (**B**). Error bars represent standard deviations (**** = *p* < 0.0001, *** = *p* < 0.001, ** = *p* < 0.01, and * = *p* < 0.05 by one-way ANOVA). Not significant represented as “ns.”

To test these hypotheses, the timing of sucrose addition was compared between AAV2.5 and AAV6, those that responded to the sugar, and AAV5, which did not. AAV2.5, AAV6, and AAV5 were added in a buffer of KPBS or KPBS with 0.5 M sucrose and added to 293T cells. To limit variables, no vectors were lyophilized for these experiments. After 30 min, the cells were washed with KPBS, and the media were replaced with media including either KPBS alone or 0.5 M sucrose KPBS of the same volume as the original AAV dose. This gave four pre- and post-infection conditions: PBS then PBS; PBS then sucrose; sucrose then PBS; and sucrose then sucrose. Figure 5A compares the fluorescence of AAV2.5-GFP-luciferase in the different conditions at 48 h post-infection, showing an increased expression when sucrose is present post-cell entry of the AAV.

Luciferase activity was assessed 48 h after infection for both AAV serotypes that responded and did not respond to sucrose, as determined by the experiments run in Figure 3. AAV2.5, AAV5, and AAV6 all had a significant increase in expression in both conditions when sucrose was added post-cellular entry. Transduction was primarily increased for AAV2.5 and AAV6, as previously observed (Figure 5B).

### 3.5. Lyophilization and Sucrose Do Not Affect the Performance of the Vector In Vivo

Given the effects of sucrose post-cell entry on AAV transduction in vitro (Figure 4), we tested if this effect could be harnessed for in vivo gene delivery. To test this, doses of 5 × 10^10^ vg of AAV2.5 GFP-luciferase in KPBS were taken from standard storage (−80 °C) and were compared to AAV2.5-GFP-luciferase in 0.5 M sucrose KPBS buffer that was lyophilized and resuspended with ddH_2_O. The prepared doses were injected intramuscularly into both quadriceps of male adult FVB mice. PBS injections were used as a negative control. After 10 days, the mice were imaged for luciferase expression using IVIS, and the radiance values were quantified (Figure S3). Under these conditions, the lyophilized AAV in 0.5 M sucrose buffer did not increase transduction over the level mediated by AAV in the standard storage buffer from −80 °C.

### 3.6. Stability of Lyophilized AAV2.5 and AAV6 at Room Temperature, 4 °C, and at −20 °C

The goal of this study was to test methods to store and deliver AAV therapies at temperatures that are less extreme than −80 °C for global distribution. For this purpose, AAV drugs would ideally be stored at room temperature. If room temperature storage is not feasible, storage in refrigerators at 4 °C would be better since most sites around the world have access to this level of refrigeration. If refrigeration did not preserve AAV stability, −20 °C freezing would be preferred over ultralow −80 °C storage.

To assess how well this simple PBS-sucrose lyophilization method might meet these needs, two different AAV serotypes, AAV2.5 and AAV6, were tested. AAV2.5 and AAV6 carrying the GFP-luciferase transgene were lyophilized in 0.5 M sucrose KPBS buffer. Poloxamer was added at 0.01% *w*/*v* to prevent adhesion to the glass vial. Each glass vial contained one “dose” of virus, or 10,000 vg per cell for one well of a 96-well plate. Stoppers were balanced on top of the vials as they were lyophilized, and the vials were capped under vacuum to stop rehydration during storage.

**Figure 6:**
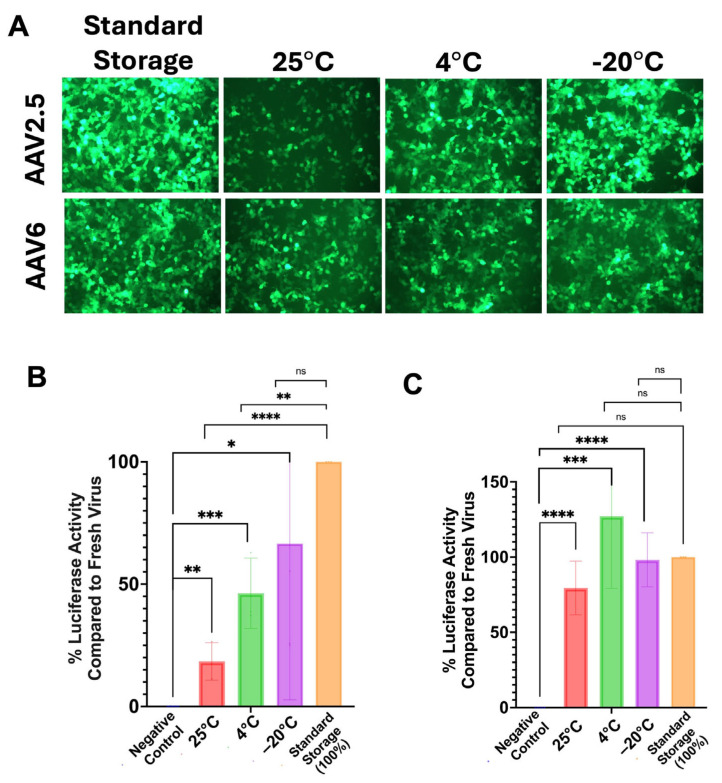
AAV responds differently depending on serotype and storage temperature. The 1:1 mixtures of AAV2.5GFP-luciferase and AAV6GFP-luciferase to buffer with 0.01% poloxamer were lyophilized in glass vials as droplets (dots), which were sealed under vacuum. After lyophilization, dots were stored at −20 °C, 4 °C, or 25 °C in sealed glass vials for 2 months in replicates of 6. At 2 months, vials were checked for seal integrity, and the intact samples were used to infect 293Ts in replicates of 4 to 6 with 10,000 vg per cell of either standard storage or lyophilized AAV2.5 of AAV6GL with buffer. Luciferase signal was assessed via luciferase assay at 48 h post-infection. Luciferase signal from each sample well was compared to the standard storage control and is reported as a percentage of the standard storage control signal. (**A**) Cell images of AAV2.5 and AAV6GL at each condition (images obtained using 10× objective), and (**B**) % activity of standard storage vector of AAV2.5 kept at 25 °C, 4 °C, or −20 °C compared to negative control/uninfected wells. Error bars represent standard deviations. (**C**) % activity of standard storage vector of AAV6 kept at 25 °C, 4 °C, or −20 °C compared to negative control/uninfected wells. Error bars represent standard deviations. Error bars represent standard deviations (**** = *p* < 0.0001, *** = *p* < 0.001, ** = *p* < 0.01, and * = *p* < 0.05 by one-way ANOVA). Not significant represented as “ns.”

After lyophilization was complete, the vials containing lyophilized virus were stored at 25 °C, 4 °C, and −20 °C for 8 weeks in replicates of six. At each time point, vials were checked for seal integrity, and those viral doses were reconstituted in water of the same volume as the original volume of virus solution that had been aliquoted in the vial. This solution was then used to infect 293T cells in a 96-well plate. GFP and luciferase activity were assessed at 48 h after infection and compared to control AAV samples that were directly thawed from −80 °C storage (Figure 6A). The control “standard storage” AAV samples were kept in the same 0.5 M sucrose KPBS poloxamer buffer and stored in the same glass vials as the lyophilized samples to account for sample loss due to adhesion. Vector luciferase activity is shown as the percentage of the luciferase signal elicited by AAV2.5 or AAV6 GFP-luciferase controls taken directly from standard storage of −80 °C (Figure 6B,C).

At two months after lyophilization, AAV2.5 and AAV6 both had significantly higher average luciferase signal than the negative control of cells treated with buffer alone for all temperature conditions at the two-month time point. AAV2.5 stored at 25 °C, 4 °C, and −20 °C produced 18%, 46%, and 67% of the −80 °C sample, respectively (Figure 6B). AAV6 stored at 25 °C, 4 °C, and −20 °C produced 79%, 127%, and 98% of −80 °C sample, respectively (Figure 6C).

## 4. Discussion

Many genetic therapies require cold-chain storage at temperatures of −20 °C to −80 °C, which can be a significant logistical issue globally. Lyophilization provides a potential solution to this issue by allowing samples to be maintained for long periods of time at less extreme temperatures. One of the most common gene therapy vectors, AAV, has previously been lyophilized, but not with a full panel of serotypes. Because of this, we set out in this study to assess the stability of a variety of serotypes of AAV after lyophilization. Through this, we additionally observed a novel interaction between sucrose and AAV serotypes 2, 2.5, and 6.

Lyophilization in 0.5 M sucrose KPBS mediated a 6-fold increase in AAV2 transduction efficiency, a 5-fold increase in AAV2.5 transduction efficiency, and a 3-fold increase in AAV6 transduction efficiency, as measured by luciferase assay. With research-grade vector production, impurities such as host–cell proteins and empty capsids may also decrease overall transduction efficiency, suggesting the vectors may maintain or benefit potency and integrity further with polishing steps utilized in GMP and clinical-level production [35,36]. Upon further study into the boost in transduction efficiency, it was determined that this phenomenon was facilitated more by sucrose, rather than by lyophilization. Spiking standard storage AAV2.5 with 0.5 M sucrose facilitated a 4-fold increase in transduction efficiency, as measured by luciferase assay. In addition, the inclusion of 0.5 M sucrose in the lyophilization buffer mediated a 6-fold increase in transduction efficiency of AAV2.5, as compared to AAV2.5 lyophilized without sucrose. The increase in transduction efficiency afforded by sucrose was so large that the transduction efficiency of AAV2.5 lyophilized in 0.5 M sucrose was 3-fold higher than standard storage AAV2.5 without sucrose. After investigating the timing of the addition of sucrose to the AAV, we concluded that the boost in transduction efficiency is not due to conformational changes allowing for easier cellular entry or receptor binding, but instead a process of post-cellular entry.

For the stability of AAV once lyophilized, the data shows that factors, including serotype and storage temperature, determine how well it will retain activity. The difference in thermostability among AAV serotypes is well-documented. The observation that AAV2.5 retains less activity than AAV6 aligns with the existing literature, which identifies AAV2—the parent capsid of AAV2.5—as the least stable among the unmodified AAV serotypes [6,8]. The modification of capsids may also decrease thermostability further, leading to AAV2.5 losing even more of its ability to retain activity through the lyophilization process and during the storage period [37]. AAV6 performed consistently better and retained almost full activity at room temperature and full activity at 4 °C and −20 °C.

An unintended condition found in both repeats of the long-term stability experiment was the status of the vial seal due to limited access to industry-level filling and stopping equipment. Vial sealing status—and by extension, hydration status—had the most pronounced impact on activity loss (Figure S2). Moisture content has been well established as a dominant factor in what drives the survival of the vector [21,38,39,40] and with an unsealed vial, the humidity was able to slowly rehydrate the sample over time.

While the in vitro data showing increased expression of AAV with sucrose buffer was promising, we wanted to know whether the effect translated into in vivo significance. If the effect was seen in vivo, there would be potential for clinical benefit for the addition of sucrose to AAV gene therapy buffers. To investigate this, we compared mice receiving AAV2.5 GFP-luciferase lyophilized in 0.5 M sucrose to the vector from standard storage intramuscularly (Figure S3). We hypothesized that a local delivery approach, like intramuscular injection, would also increase the ability of the sucrose to directly affect the AAV entry and post-entry into the transduced cells. At 10 days post-injection, there was no significant difference between the AAV in PBS and the AAV in 0.5 M sucrose in KPBS (Figure S3). We hypothesize that to see the benefit of the sucrose, the delivery may need a scaffold or an alternative way to keep the buffer local to the site of vector transduction.

This work demonstrates that multiple AAV serotypes can be stabilized for global shipment and storage. The stability differences between serotypes in longer-term studies highlight the potential considerations that may need to be considered during clinical translation. However, the hydration of the sample is not likely to be an issue in commercial-level studies, leading to better stability in multiple temperature conditions. The ability to store AAV vectors at 4 °C after lyophilization would be a major improvement in the storage parameters required for genetic therapies and would be a step forward in both expanding global accessibility and extending their shelf life, each a crucial factor for distribution and clinical applications [10,41]. The increased stability also reduces logistical challenges and operational costs, making AAV-based gene therapies more accessible [10,21]. Beyond stability, lyophilization also allows for the creation of stable, ready-to-use dosage forms that can be reconstituted with a suitable solvent before administration. This enhances the practicality and usability of AAV vectors in clinical settings, where rapid reconstitution is often required [41].

## Figures and Tables

**Table 1:** Lyophilization protocol for AAV [19].

Temperature (°C)	Time (h)
−40	2
−38	11
−10	2
0	2
25	3

## Data Availability

The original contributions presented in this study are included in the article/Supplementary Material. Further inquiries can be directed to the corresponding author.

## Supplementary Materials

The following supporting information can be downloaded at: https://www.mdpi.com/article/10.3390/biomedicines14010025/s1, Figure S1. AAV2.5 Transduction Efficiency After Lyophilization on Different Cell Types. AAV 2.5 expressing GFP-Luciferase (AAV2.5GL) was combined in a 1:1 mixture of 1M Sucrose KPBS buffer to virus and lyophilized. Lyophilized virus compared with non-lyophilized virus freshly thawed from the −80C freezer (standard storage) was used to infect 293T cells in triplicate at a multiplicity of infection (MOI) of 10,000 vg per cell. (A) Luciferase assay was performed on the cells 72 hours post-infection with either Standard Storage or Lyophilized virus compared with uninfected controls. (B) This phenomenon was also observed in CHO-Jam1 cells. Error bars represent standard deviations (**** = *p* < 0.0001, * = *p* < 0.05 by one way ANOVA). Figure S2. Effect of Vial Seal Status on Transduction Efficiency. A) % activity of standard storage vector of each serotype at each temperature condition with unsealed vials included. Only AAV6GL at 4°C had no unsealed vials. At time of rehydration, the vial’s sealing status was noted via whether vacuum held for the duration of the study. Error bars represent standard deviations (** = *p* < 0.01, * = *p* < 0.05 by one way ANOVA). Figure S3. Effects of Lyophilization and Sucrose on AAV in vivo. To examine the effect of sucrose in vivo, AAV2.5-GFPLuciferase was lyophilized with 0.5M sucrose and was compared freshly thawed AAV from the –80C and PBS alone given intramuscularly to FVB mice in the right and left quadriceps muscle at a dose of 5e10vg (A). Luciferase expression was measured via IVIS imaging at 10 days after injection (B). (Created in BioRender. Mcglinch, E. (2025) https://BioRender.com/vwqsu4l).
