# Supplementary material for: Lyophilization of Adeno-Associated Virus Serotypes for Storage and Global Distribution
Source: Biomedicines. 2025 Dec 22;14(1):25. doi: 10.3390/biomedicines14010025 (PMC12838101; doi:10.3390/biomedicines14010025)
Supplement: Supplementary file 1 [file biomedicines-14-00025-s001.zip › Supplemental Figures.pdf]

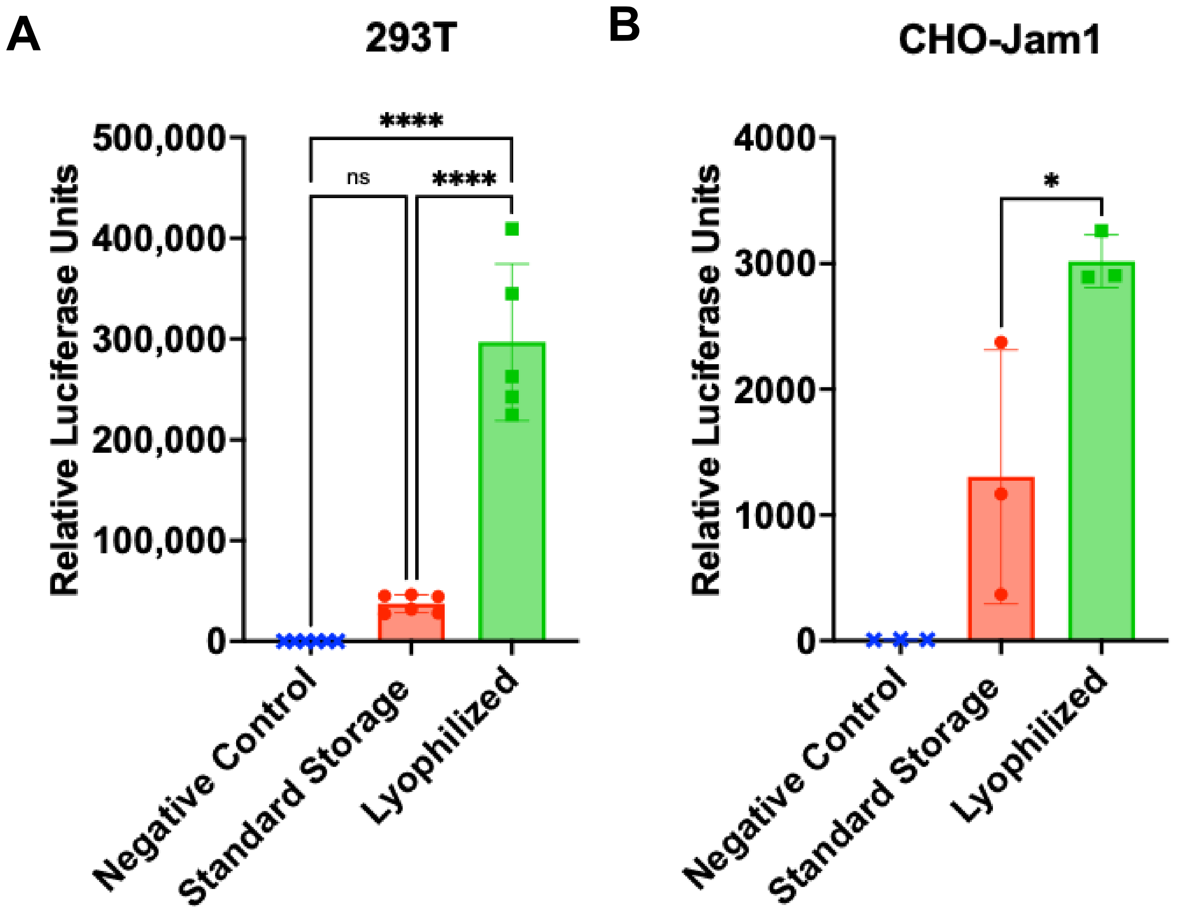

Figure S1. AAV2.5 Transduction Efficiency After Lyophilization on Different Cell Types. AAV 2.5 expressing GFP-Luciferase (AAV2.5GL) was combined in a 1:1 mixture of 1M Sucrose KPBS buffer to virus and lyophilized. Lyophilized virus compared with non-lyophilized virus freshly thawed from the –80C freezer (standard storage) was used to infect 293T cells in triplicate at a multiplicity of infection (MOI) of 10,000 vg per cell. A) Luciferase assay was performed on the cells 72 hours post-infection with either Standard Storage or Lyophilized virus compared with uninfected controls. B) This phenomenon was also observed in CHO-Jam1 cells. Error bars represent standard deviations (**** = $p<0.0001$, * = $p<0.05$ by one way ANOVA).

A

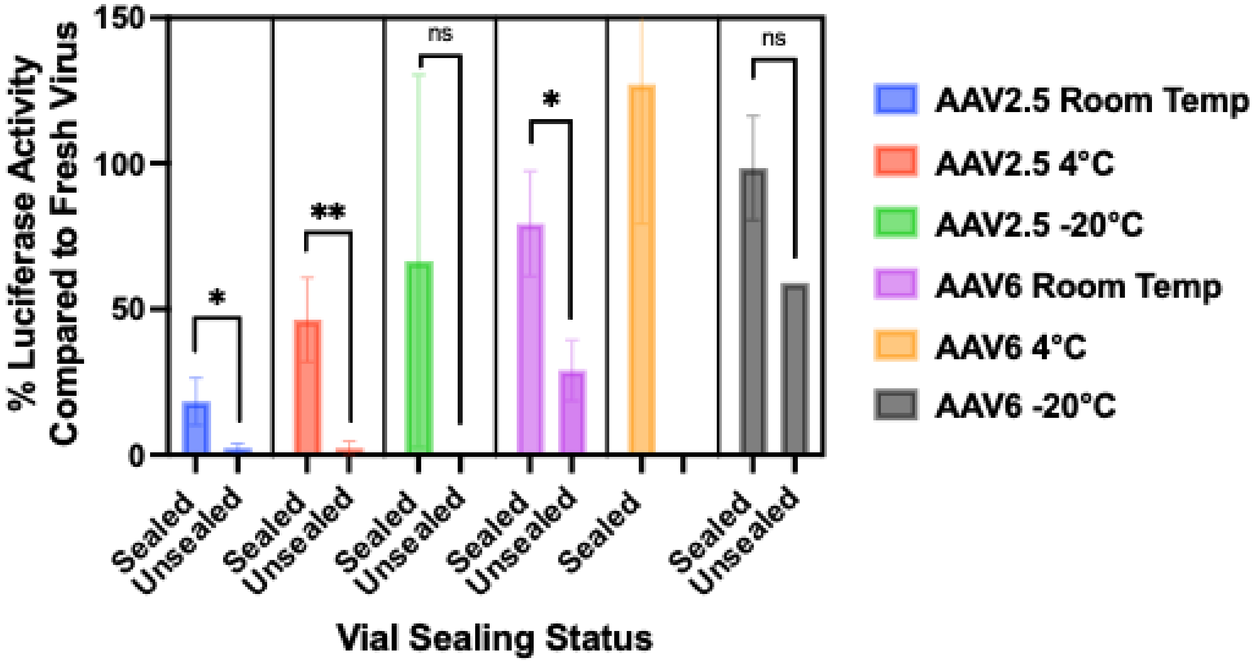

Figure S2. Effect of Vial Seal Status on Transduction Efficiency. A) % activity of standard storage vector of each serotype at each temperature condition with unsealed vials included. Only AAV6GL at 4°C had no unsealed vials. At time of rehydration, the vial's sealing status was noted via whether vacuum held for the duration of the study. Error bars represent standard deviations (** = $p<0.01$, * = $p<0.05$ by one way ANOVA).

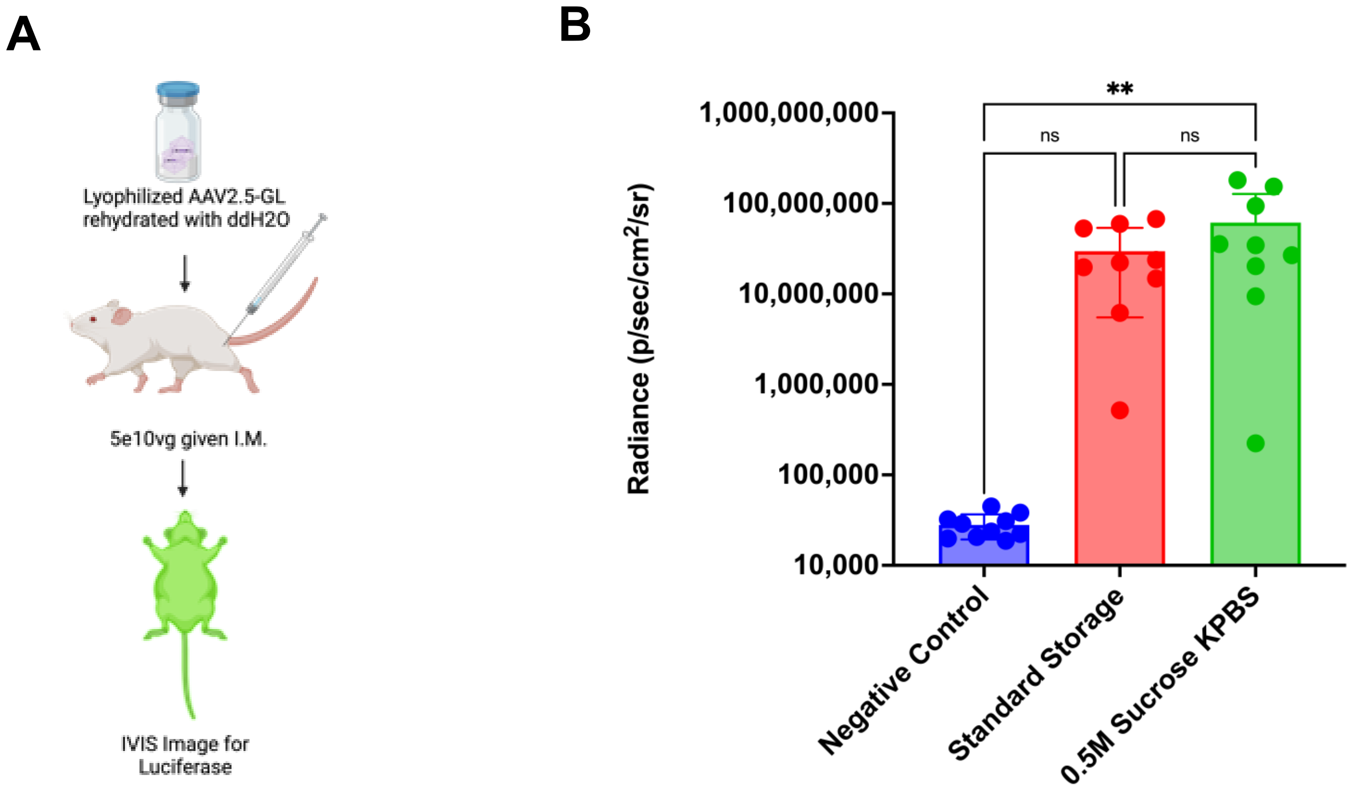

Figure S3 Effects of Lyophilization and Sucrose on AAV in vivo. To examine the effect of sucrose in vivo, AAV2.5-GFPLuciferase was lyophilized with 0.5M sucrose and was compared freshly thawed AAV from the –80C and PBS alone given intramuscularly to FVB mice in the right and left quadriceps muscle at a dose of 5e10vg (A). Luciferase expression was measured via IVIS imaging at 10 days after injection (B). (Created in BioRender. Mcglinch, E. (2025) https://BioRender.com/vwqsu4l)
